# Advancing ovarian folliculometry with selective plane illumination microscopy

**DOI:** 10.1038/srep38057

**Published:** 2016-12-01

**Authors:** Hsiao-Chun Amy Lin, Rahul Dutta, Subhamoy Mandal, Alexander Kind, Angelika Schnieke, Daniel Razansky

**Affiliations:** 1 Institute for Biological and Medical Imaging, Helmholtz Zentrum München, Ingolstädter Landstraße 1, 85764 Neuherberg, Germany; 2 Faculty of Medicine, Technische Universität München, Ismaningerstraße 22, 81675 Munich, Germany; 3 Chair of Livestock Biotechnology, Technische Universität München, Liesel-Beckmann Straße 1, 85354 Freising, Germany; 4 Chair for Biological Imaging, Faculty of Electrical Engineering and Information Technology, Technische Universität München, Arcisstraße 21, 80333 Munich, Germany

## Abstract

Determination of ovarian status and follicle monitoring are common methods of diagnosing female infertility. We evaluated the suitability of selective plane illumination microscopy (SPIM) for the study of ovarian follicles. The large field of view and fast acquisition speed of our SPIM system enables rendering of volumetric image stacks from intact whole porcine ovarian follicles, clearly visualizing follicular features including follicle volume and average diameter (70 μm–2.5 mm), their spherical asymmetry parameters, size of developing cumulus oophorus complexes (40 μm–110 μm), and follicular wall thickness (90 μm–120 μm). Follicles at all developmental stages were identified. A distribution of the theca thickness was measured for each follicle, and a relationship between these distributions and the stages of follicular development was discerned. The ability of the system to non-destructively generate sub-cellular resolution 3D images of developing follicles, with excellent image contrast and high throughput capacity compared to conventional histology, suggests that it can be used to monitor follicular development and identify structural abnormalities indicative of ovarian ailments. Accurate folliculometric measurements provided by SPIM images can immensely help the understanding of ovarian physiology and provide important information for the proper management of ovarian diseases.

The incidence of ovarian ailments such as premature ovarian failure (POF) and polycystic ovary syndrome (PCOS) has risen in recent years, constituting a major cause of female subfertility in the modern world^1^,^2^. Determination of ovarian status and follicle monitoring are important first steps in evaluating infertile females, making ovarian imaging the most common diagnostic approach for female infertility. The ovary is imaged for morphology (normal or polycystic), abnormalities (e.g. cysts, dermoids, endometriomas, tumours), follicular growth in ovulation monitoring, and for evidence of ovulation and corpus luteum formation and function^3^. Advances in diagnostic imaging technologies such as pelvic MRI and ultrasound have provided new insights into the human reproductive system. Yet, the imaging contrast and spatial resolution of clinical imaging methods are far inferior to those routinely obtained with optical microscopy. Histological evaluation of *ex vivo* samples thus remains of indispensable value for diagnosis and research. However, standard *ex-vivo* histological analyses of ovarian follicles suffer the depth limitation inherent in traditional microscopy. Physical sectioning is also labour- and time-intensive, and may destroy key morphological features in the samples.

The diameter of the ovarian follicle and the developing oocyte within, and the thickness of the follicular wall are important parameters for clinical management of ovarian ailments. A normal mammalian ovary harbours 25,000-10^6^ primordial follicles at puberty. A sketch illustrating the various stages is shown in Fig. 1a. The earliest stage of follicular growth is the primordial follicle, where the oocyte is arrested in the last stage of prophase and is surrounded by a single layer of granulosa cells. Hormonal stimulation causes the oocyte to enlarge, and follicular cells to divide; a follicle with two layers of follicular cells is called a primary follicle. During the follicular phase, a small cohort of follicles (approximately 10–14) begins to develop. In uniparous mammals, one follicle is physiologically selected to ovulate, exhibiting greatly increased hormonal activity and increased growth^4^, while the others undergo atresia. The primary follicle transitions through the secondary follicle stage to develop into a Graafian follicle, while the oocyte completes the first meiotic division and becomes a secondary oocyte. Upon ovulation, the oocyte begins its second meiotic division up to metaphase II, which continues only if it is fertilised. Follicles destined to ovulate display different morphological characteristics than those destined for atresia, and these are observable days before physiological selection becomes apparent. Measurement of the follicle wall is useful because walls of follicles destined to ovulate are thinner^5^. However, evaluation by the commonly used ultrasonography reveals the growing follicles only as black bubble-like features. Most folliculometric information, including follicular wall thickness, is determined by manual or semi-automated segmentation.

The study we describe explore the use of selective plane illumination microscopy (SPIM) to gain accurate and informative folliculometric data from scanned ovaries. SPIM is a relatively new technology that is increasingly used because it enables rapid visualisation of both chemically-cleared and living specimens^6^,^7^,^8^. A single plane of the sample is optically excited by a thin light sheet and 2D fluorescent images captured by a camera placed orthogonally to the light sheet. Translation of either the light sheet or the sample allows the assembly of 3D volumetric image stacks. The sample remains intact, allowing repeated imaging from multiple angles, to increase the quality of the combined 3D rendering. Any reduction in image quality due to physical destruction of the tissue is also avoided. SPIM is time- and labour-efficient and well suited for producing high resolution imaging stacks of 3D biological samples. It has been used for *in vivo* studies of naturally transparent biological specimens, such as zebrafish embryos and *Drosophila* pupae^9^,^10^, as well as samples rendered optically transparent by chemical clearing^11^,^12^. It was hypothesized that it may prove to be useful as a supplementary tool additional to the current conventional methods for ovarian studies. Pig was chosen as the model organism for this study because of its similarity to humans in terms of genetics, anatomy, and physiology.

## Results and Discussion

**Figure 1:**
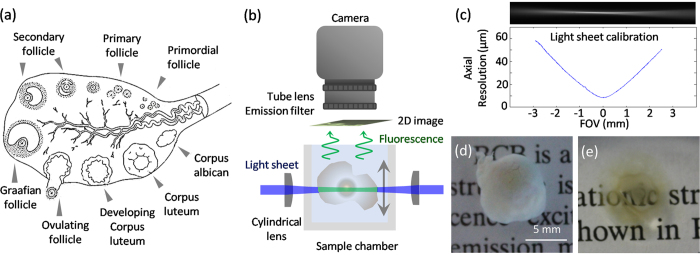
Ovarian follicles and SPIM experimental setup. (**a**) Representative sketch of follicle anatomy. Primordial follicles are small, and usually located close to the outer edge of the ovarian cortex surrounded by a single layer of granulosa cells. When the primordial follicle receives hormonal stimulation it forms a primary follicle with two layers of granulosa cells. The primary follicle transitions through the secondary follicle stage, during which small amounts of fluid accumulate in the intracellular spaces. These gradually coalesce to form an antrum, and later the Graafian follicle forms. In uniparous mammals one Graafian follicle ovulates, while the rest degenerate into atretic follicles. Upon pregnancy, the ovulating follicle forms the corpus luteum. The figure was produced, in part, using Servier Medical Art. (**b**) Side view of the SPIM imaging system. Two light-sheets were spatially overlapped and oriented horizontally. The imaging camera and the imaging optics were placed directly above the sample, which was centrally oriented at the beam waists of the light sheets. By placing the camera orthogonally to the light sheets, 2D fluorescent images through the entire specimen measuring up to 10 mm across could be captured. The samples were placed inside a glass chamber filled with clearing solution and scanned in a linear (vertical) geometry. By translating either the light-sheet or the sample chamber, full 3D image stacks of the sample could be obtained. (**c**) Light sheet characterisation using a solution of fluorescent dye. The axial resolution across the field-of view (FOV) is determined by the light sheet thickness of 16 μm at the beam waist, expanding to about 60 μm at a distance of 3 mm away from the centre of FOV. (**d**) Brilliant Cresyl Blue (BCB) stained ovarian follicle. (**e**) Sample after clearing. Ethanol was used for dehydrating the sample, and 2:1 benzyl alcohol/ benzyl benzoate (BABB) for clearing.

An SPIM system was built in-house to optimally support the imaging of ovarian follicles. Figure 1b shows a side-view of the SPIM system, and its characterization for the large field of view (FOV) is presented in Fig. 1c. Small clusters of ovarian follicles were dissected (Fig. 1d) and chemically cleared (Fig. 1e). For more detailed descriptions of the imaging system and the sample preparation procedure please refer to the Methods section.

**Figure 2:**
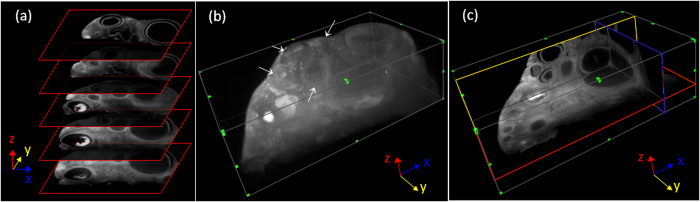
Three-dimensional renderings of an intact sample. (**a**) As the sample is translated through the light-sheet along the z direction, a series of images are recorded. The images shown are of an ovarian follicle sample, spaced with intervals of 350 μm. (**b**) MIP of the 3D reconstruction obtained from the image stack, containing the full volumetric anatomical information. Outlines of four individual follicles can be identified in this view, indicated by white arrows. (**c**) Single plane images in each x (blue), y (yellow), and z (red) dimension is shown. The reslicing in x and y dimensions was done using a uniform grid, with the red slice indicating the imaging plane.

As the sample is translated through the light-sheet, a series of images were recorded. In Fig. 2a the sample is an ovarian follicle cluster, and the images are uniformly spaced with intervals of 350 μm. A 3D reconstruction can be obtained from the imaging stack, and Fig. 2b shows the maximum intensity projection (MIP). The white arrows indicate the outlines of four individual follicles. Containing full anatomical information, the volumetric data can be further resliced in the x and y directions. From the full volumetric reconstruction, reslicing in the x and y dimensions was possible. Figure 2c shows a single plane image from each dimensions (x: blue, y: yellow, z: red), resliced using uniform grids and displayed with isotropic scaling, with the red box indicating the imaging plane.

**Figure 3:**
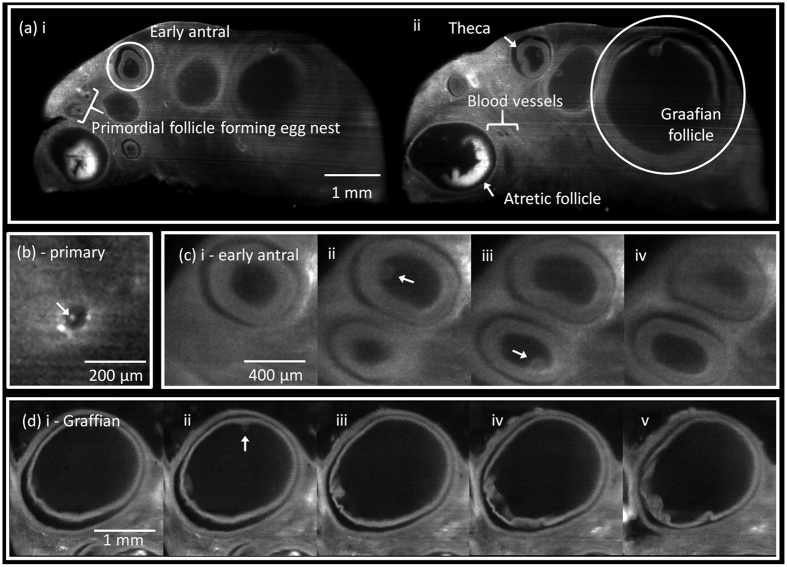
SPIM imaging of ovarian follicles. (**a**) Full FOV cross-sectional SPIM images taken at two different planes of a cleared follicle cluster. A few features are indicated: (i) early antral follicle and primordial follicle forming egg nest; (ii) Graafian and atretic follicles, theca layer, and blood vessels. (**b**) Primary follicle measuring 74 μm in diameter. The arrow indicates the COC, which was found to be 29 μm in diameter. (**c**) Early antral follicles with average diameters of 586 μm (top) and 476 μm (bottom), shown in subsequent imaging planes with uniform separation of 64 μm. Arrows indicate the COCs with diameters of 101 μm (top) and 98 μm (bottom). (**d**) Graafian follicle with average diameter of 1,788 μm, shown in subsequent imaging planes with uniform separation of 98 μm. The arrow indicates a COC with a diameter of 104 μm.

Single plane SPIM images of typical cleared ovarian follicles are shown in Fig. 3. The outer cortex of the ovary consists of ovarian follicles interspersed by stroma. Each follicle is composed of cumulus oophorus, membrana granulosa, corona radiata, and a developing oocyte surrounded by the zona pellucida. We imaged all samples in their entirety, as their sizes matched the camera FOV. A wide range of individual follicles was extracted from two different sets of image stacks. Figure 3a shows single image slices taken at two different planes of the same sample. The follicular antrum and *theca interna* layers are visible, allowing individual follicles to be readily identified. Follicles of all sizes can be seen, and additional morphological details such as atretic follicles, blood vessels and interstitial connective tissue between adjacent follicles could be identified. In the single plane images, the primordial follicles may seem similar to blood vessels. They are however easily distinguishable when examined in the entire image stack of the sample due to their dramatically different anatomy. Supplementary Movie 1 shows a ‘fly-through’ of the entire sample image stack. The elongated geometry of blood vessels can be seen extending throughout the sample stack, while follicles appear and disappear from the images much faster. The high contrast (signal-to-noise ratio) and image resolution achieved by SPIM allow for easy discrimination of the different anatomical features. SPIM images were analysed for the most clinically or diagnostically relevant morphological features, specifically: volume and spherical asymmetry of follicles, diameter of the developing cumulus oophorus complexes (COC), and follicle wall thickness.

Primordial follicles often form clusters, termed the egg nest, as shown in Fig. 3a. Primordial follicles form the ovarian reserve and are extremely important for reproductive health, hence the motivation to develop methods of counting follicles and thus ascertaining reproductive age. In humans at 18–22 weeks post-conception, the cortex of the ovary typically contains 4 to 5 million primordial follicles; this number decreases throughout reproductive life, falling to as low as 1,000 at menopause^4^. However, non-destructive imaging methods such as ultrasound cannot visualise primordial follicles due to insufficient resolution.

Individual follicles were identified, and categorized by three main stages of folliculogenesis: primary, antral, and Graafian. Figure 3b shows an SPIM image of a primary follicle, measured diameter 74 μm, with a primary oocyte. Antral follicle diameters typically measure between 100 μm (early antral) and 700 μm. Figure 3c shows two early antral follicles: 590 μm (top) and 480 μm (bottom) in diameter. These follicles show a clear antral cavity surrounded by a smooth *theca interna* layer clearly defining the follicle boundaries. COCs can be seen in panels (ii) and (iii). Figure 3d shows a 1.8 mm (diameter) Graafian follicle, and on panel (ii) the COC can be clearly seen attached to the inner follicle wall. COCs were estimated to be detectable in approximately 70% of the follicles, and the COC diameters varied between 40 μm and 110 μm. Detection of COCs as small as 40 μm in diameter approaches the averaged 25 μm obtained by histological analysis^13^. It is worth noticing that putative COCs appearing as protrusion into the fluid-filled lumen can only be detected in less than 25% of the ovarian follicles by ultrasound biomicroscopy. SPIM far outperforms the ultrasound techniques in terms of the spatial resolution^14^, and is thus more suitable for small follicle detection.

Accurate estimation of the follicle diameter is complicated due to their irregular shape. Typically, conventional analysis of histological images was carried out by taking two orthogonal measurements intersecting at the midpoint of the first measurement, and the averaged value taken as the follicle diameter^13^. An estimation of the follicle volume is then made, and accordingly, the developmental stage of the follicle is calculated. However this method involves certain assumptions: the average of only two measurements is a sufficient estimation of the follicle diameter in the imaging plane; the follicle’s shape irregularities in other planes are negligible; the follicle has been sectioned perfectly in the middle plane; and physical sectioning has not deformed the anatomy. By discounting all of these factors, errors can incur.

**Figure 4:**
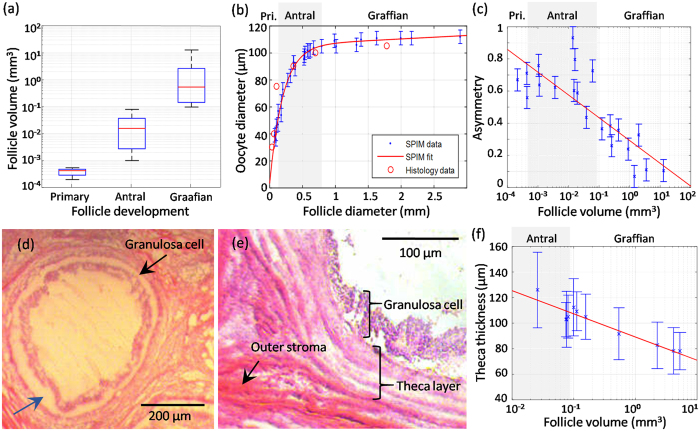
Quantitative morphological analysis across stages of folliculogenesis. (**a**) Follicle volume versus developmental stage: statistical analysis of follicle volume for primary (4.4 × 10^−4^ [3.0 × 10^−4^–5.0 ×  10^−4^] mm^3^), antral (1.6 × 10^−2^ [2.9 × 10^−3^–3.8 × 10^−2^] mm^3^), and Graafian (0.54 [0.15–2.63] mm^3^) follicles. (**b**) Quantification and validation of the oocyte diameter versus the follicle average diameter. Our results derived by SPIM are labelled in blue while the histological values reported in literature are shown by red circles. The adjusted coefficient of determination (R-squared) between the literature values and the SPIM-fitted curve was 0.76, indicating good agreement. (**c**) Normalized asymmetry of 21 follicles across a range of developmental stages, determined by the major-minor axes ratio of the follicle equatorial cross-section. An inverse relationship can be observed when presenting the volume on a logarithmic scale, indicating follicles approaching spherical shape at more advanced developmental stages (R^2^ = 0.65). (**d**) H&E validation of a cleared follicle made after SPIM imaging. The black arrow indicates the granulosa cells, and the blue arrow indicates an area confirming the delamination of the granulosa layer from the thecal layer. (**e**) H&E close-up of the follicle boundary, clearly separating into the granulosa cell, the theca layer, and the stroma. Here, the sample was not cleared, and no delamination was present. (**f**) Theca wall thickness versus follicle volume measured from the SPIM data. 10 antral and Graafian follicles from two different samples were analysed. The averages are plotted along with the standard deviations as error bars. Results indicate a clear inverse correlation between the wall thickness and follicle volume when plotting the latter on a logarithmic scale (R^2^ = 0.84).

On the other hand, follicle volume would be a more robust parameter for characterizing the size of these asymmetric objects. For this, complete stacks of individual follicles were segmented, allowing for high precision measurement of each follicle volume. Statistical analysis of the average and interquartile range of the follicle volume for primary (4.4 × 10^−4^ [3.0 × 10^−4^–5.0 × 10^−4^] mm^3^), antral (1.6 × 10^−2^ [2.9 × 10^−3^–3.8 × 10^−2^] mm^3^), and Graafian (0.54 [0.15–2.63] mm^3^) follicles are presented in Fig. 4a. As high-resolution information in all planes was herein made full use of, all anatomical aberrations in every plane of the follicles are considered.

To validate the quantitative method, SPIM results were compared to histological statistics. Since follicle diameters were reported in the literature, we estimated the average follicle diameters in our measurements from the follicle volumes by assuming spherical symmetry, yielding values varying from 74 μm to 2.9 mm. Figure 4b depicts the oocyte size related to follicle diameter, with the red circles representing porcine ovarian follicle diameters reported in literature^13^. A total of 30 data points were taken from the SPIM data from two different samples, as plotted in blue. The middle planes of the COCs where used to perform the diameter estimations. The image stacks were inspected carefully to ensure that the equatorial plane of the COCs was selected to avoid any underestimation. The adjusted coefficient of determination (R-squared) between the literature values and the SPIM-fitted curve was 0.76, indicating good agreement.

We further analyzed the follicle shape asymmetry by approximating the follicle geometry by an ovoid, and selecting the image plane having the maximum follicle area as the equatorial plane. The normalized ratios of the major and minor axes were then defined as the asymmetry parameter. The results encompassing measurements from 21 follicles across a range of developmental stages are plotted in Fig. 4c. An inverse relationship can be seen, indicating that follicles approach a spherical shape with advanced development (R^2^ = 0.65).

Another important feature of the SPIM images was the distinct visualisation of the follicular thecal walls. During the clearing phase there was delamination of the granulosa layer from the thecal layer, delineated by a black area in the SPIM images. While this may induce discrepancy between the anatomy pre- and post-clearing, estimated to be <4% in measured diameter, the overall methodology proved to be particularly advantageous in clearly demarcating the thecal layer from the granulosa layer. A series of H&E sections was also performed on the cleared follicles after SPIM imaging to confirm the thecal boundary, shown clearly in Fig. 4d. The black arrow indicates the granulosa cells, while the blue arrow indicates an area demonstrating the delamination of the granulosa layer from the thecal layer. A close-up of a follicle is shown in Fig 4e, where the partial view of the boundary can be clearly sectioned into granulosa cell, theca layer, and outer stroma. Here, the sample was not cleared, and no delamination was present. Contraction of samples has been widely observed with the BABB clearing protocol, which includes dehydration steps using ethanol. While this effect has been negligible (~4%) in this work, it is still more likely that the delamination is due to shrinking of granulosa rather than expansion of the thecal layer.

The thecal wall contains the secretory cells responsible for the production of fluid contained by the wall. Thecal wall thicknesses of 10 early antral and Graafian follicles from two samples were measured. Wall thickness is however not uniform, so a distribution was measured for each follicle. To avoid over-estimating the theca thicknesses, the 2D image with the maximum follicle cross-section area was selected to ensure the analysis is conducted on the equatorial plane. For each pixel along the outer edge of the theca layers, the shortest distance to the inner edge was found. The number of measurements taken per image plane varied from ~300 to ~2,000 for different antral follicle sizes.

Figure 4f plots the theca thickness means along with their respective standard deviations as error bars for each follicle analysed. An inverse correlation between follicle diameter and thecal wall thickness is again observed (R^2^ = 0.64). For the largest Graafian follicle, wall thicknesses were around 90 μm, while the antral follicle walls were around 120 μm, indicating that variations in the follicle wall thickness may additionally serve for the characterization of follicle development^5^.

While SPIM cannot fully replace the traditional methodology used for ovarian studies, it has unique advantages and compatibility with this specific size and type of samples to offer complementary information for biological studies. Ultrasonic biomicroscopy is conventionally used for non-invasive monitoring of ovarian follicles, with the main advantage of *in vivo* application, but the images have lower contrast and poorer resolution than the SPIM images shown here. Ultrasound fails to detect primordial or primary follicles in the ovary, and can seldom detect the growing COC in Graafian follicles^15^. Although SPIM cannot be used for *in vivo* biological studies, it offers a level of morphological detail that may greatly aid developmental studies. Here, the smallest anatomical feature we could image was ~10 μm, which is supported by the 6 μm in-plane resolution offered by our SPIM system.

H&E staining is the current gold standard method for anatomical ovarian investigation. While this imaging modality may offer better spatial resolution, the required sample destruction due to slicing leads to the inevitable decrease in useful morphological information. On the other hand, SPIM offers key practical advantages over classical histology as no physical slicing is required. Not only does this preserve valuable morphological information, but imaging of intact samples is faster and requires less effort. Multi-slice data acquisition by SPIM is a fast and automated procedure, while the large FOV additionally offers full coverage of any follicle clusters up to 10 mm in size in their entirety. The full diameter within the sample is then imaged in a single 2D image acquisition, and when combined with a high-speed camera and a motorised sample translation stage, a short imaging time of a few minutes can be achieved.

## Conclusions

We describe a novel combination of clearing and imaging porcine ovarian follicles with SPIM. The follicular antrum and *theca interna* layers were distinctly visible, allowing individual follicles to be easily identified. Follicles of all developmental stages were identified and their volume and asymmetry characterised, ranging from small primordial follicles up to 2.5 mm Graafian follicles. Clearly distinguishable COCs protruding into the follicular antrum of several antral and Graafian follicles were measured at 40–110 μm, and their correlation with the developmental stage of the follicles agreed well with the literature, indicating good quantitative capacity of the method. The first distribution measurements of *theca* thickness for follicles of different developmental stages were conducted, yielding results ranging from 90 to 120 μm. We have also found an inverse correlation between follicle asymmetry and their developmental stage as well as follicle diameter and thecal wall thickness.

In comparison to ultrasound biomicroscopy, the superior spatial resolution and high contrast offered by SPIM provide better quantification of the different anatomical features. The unique capability to image 3D follicle anatomy while retaining morphological integrity, coupled with ease and speed, further makes it superior to conventional H&E histology. SPIM was further shown to be suitable for characterising the developmental stages of ovarian follicles, without the need to aspirate oocytes. Abnormalities in the physical dimensions could indicate possible ovarian diseases, and precise folliculometric measurements will be of immense importance in improving the understanding of ovarian physiology and developing new applications to better manage ovarian diseases.

## Materials and Methods

Animal experiments were approved by the Government of Upper Bavaria (permit number 55.2-1-54-2532-34-09) and performed according to the German Animal Welfare Act and European Union Normative for Care and Use of Experimental Animals.

### Selective-plane illumination microscopy (SPIM) system

An SPIM system was built in-house to optimally support imaging of ovarian follicles. The light source was an 80 mW continuous wave diode-pumped solid-state (DPSS) laser at 670 nm (Frankfurt laser company, Germany), with a beam quality factor, M^2^, of 1.10. A 5X telescope system was used to expand the beam diameter to 10 mm to allow excitation of the full width of the ovary samples. A beam splitter divided the main beam to allow double-sided illumination and reduce the effects of light attenuation due to absorption and scattering when imaging through large samples.

Figure 1b shows a side-view of the SPIM system. Thin sheets of light were generated using cylindrical lenses with focal lengths of 40 mm. The light-sheets were oriented horizontally, with the sample centred at the beam waist. To capture 2D fluorescent images, a 5-MP scientific complementary metal oxide semiconductor (sCMOS) camera (Model: pco.edge, PCO AG, Kelheim, Germany) was placed directly above the sample, facing downwards. The high-end scientific camera allows acquisition of up to 100 frames per second with 2,560 × 2,160 pixel resolution and high sensitivity supported by a dynamic range of 14 bits and 1.0 e- readout noise. An EC Epiplan-Neofluar objective (Zeiss, Germany) with a magnification of 2.5X and a NA of 0.06 was used because it provided a large working distance of 35 mm, and 220 μm depth of field. Fluorescence signals were filtered using a bandpass fluorescence filter centred at 690 nm with 10 nm FWHM (Chroma, USA), and a tube lens was used to focus the fluorescent image onto the camera. The lateral resolution of the imaging system was characterised using line pair targets, and was determined to have an effective FOV of 10 × 9 mm with <6 μm lateral resolution. The light sheets were characterised by rotating the cylindrical lens by 90° and filling the sample chamber with fluorescent dye Alexa Fluor 488 (Life Technologies, USA) diluted in deionised water, which allowed the light sheets to be imaged by the camera, as shown in Fig. 1c. The axial resolution was defined by the light-sheet thickness, and was found to be 11 μm at the beam waist expanding to about 60 μm at a 3 mm distance from the centre of the FOV.

### Ovary samples

Ovaries from prepubertal gilts were brought from a local slaughterhouse within one hour of collection in a temperature-controlled box maintained at 39 °C. Due to the FOV limitation of the SPIM system, individual follicles of 5–8 mm diameter and small groups of follicles were dissected out using ultrafine surgical blades. The samples were stained with Brilliant Cresyl Blue (BCB)^16^, then chemically cleared using an alcohol-based method^11^,^12^. Follicles were dehydrated using 50%, 80%, and 100% ethanol for 12 hours each, repeating the last step twice, then cleared in 2:1 benzyl alcohol/benzyl benzoate (BABB) solution for 6 hours. This rendered the samples optically transparent and ready for imaging. Figure 1d and e show a sample before and after clearing.

### SPIM imaging

Samples were fixed on a holder and placed inside the sample chamber. A linear motorised stage (MTS25/M, Thorlabs, USA) was used to translate the sample vertically, allowing different planes to be excited by the light sheet. Rapid translation enabled stacks of up to 600 cross-sectional SPIM images to be collected within two minutes. The sample was translated by ~16 μm between successive images. During imaging, the sample chamber was filled with the clearing solution BABB to match the refractive index of the medium to the sample, avoiding any image distortion. A glass sample chamber was used to avoid any reaction with the clearing solution. Image analyses to extract morphological measurements were carried out using ImageJ software. For quantitative analysis of the follicle volume and theca thickness, individual follicles and theca layers were segmented manually. Haematoxylin and eosin (H&E) stained porcine ovary sections were used as standards for comparison.

## Additional Information

**How to cite this article**: Lin, H.-C. A. *et al*. Advancing ovarian folliculometry with selective plane illumination microscopy. *Sci. Rep.* **6**, 38057; doi: 10.1038/srep38057 (2016).

**Publisher's note:** Springer Nature remains neutral with regard to jurisdictional claims in published maps and institutional affiliations.

## Supplementary Material

Supplementary Information

Supplementary Video
