# Supplementary material for: Advancing ovarian folliculometry with selective plane illumination microscopy
Source: Sci Rep. 2016 Dec 1;6:38057. doi: 10.1038/srep38057 (PMC5131314; doi:10.1038/srep38057)
Supplement: Supplementary Video [file srep38057-s2.pdf]

Advancing ovarian folliculometry with selective plane illumination microscopy

Hsiao-Chun Amy Lin[1,2+], Rahul Dutta[3+], Subhamoy Mandal[1,4], Alexander Kind[3], Angelika Schnieke[3], Daniel Razansky[1,2*]

[1] *Institute for Biological and Medical Imaging, Helmholtz Zentrum München, Ingolstädter Landstraße 1, 85764 Neuherberg, Germany*

[2] *Faculty of Medicine, Technische Universität München, Ismaningerstraße 22, 81675 Munich, Germany*

[3] *Chair of Livestock Biotechnology, Technische Universität München, Liesel-Beckmann Straße 1, 85354 Freising, Germany*

[4] *Chair for Biological Imaging, Faculty of Electrical Engineering and Information Technology, Technische Universität München, Arcisstraße 21, 80333 Munich, Germany*

[+] equal contribution

* Corresponding author: dr@tum.de

Supplementary Information

Supplementary Movie 1

SPIM 'fly-through' of an ovarian follicle cluster sample. Follicles of all sizes can be seen, and additional morphological details such as atretic follicles, blood vessels, and interstitial connective tissue between adjacent follicles could be identified.
